# Very low‐amplitude muscle activity increases probability of motor evoked potentials in healthy individuals and in amyotrophic lateral sclerosis

**DOI:** 10.1113/EP093429

**Published:** 2026-07-05

**Authors:** Séamus O'Sullivan, Yasmine Tadjine, Gabriel R. Palma, Narin Suleyman, Eva Woods, Antonio Fasano, Cathal Walsh, Friedemann Awiszus, Orla Hardiman, Richard G. Carson, Roisin McMackin

**Affiliations:** ^1^ Discipline of Physiology, School of Medicine, Trinity College Dublin The University of Dublin Dublin Ireland; ^2^ Academic Unit of Neurology, School of Medicine, Trinity College Dublin The University of Dublin Dublin Ireland; ^3^ Department of Neurology Beaumont Hospital Dublin Ireland; ^4^ TCD Biostatistics Unit, Public Health and Primary Care, School of Medicine, Trinity College Dublin The University of Dublin Dublin Ireland; ^5^ Department of Orthopaedic Surgery Otto‐von‐Guericke University Magdeburg Germany; ^6^ Trinity College Institute of Neuroscience, Trinity College Dublin The University of Dublin Dublin Ireland; ^7^ School of Psychology, Trinity College Dublin The University of Dublin Dublin Ireland

**Keywords:** amyotrophic lateral sclerosis, resting motor threshold, transcranial magnetic stimulation

## Abstract

Muscle contraction increases motor evoked potential (MEP) amplitude, decreasing motor threshold (MT). Correspondingly, trials where baseline EMG amplitude exceeds a specified threshold are often rejected. We aimed to investigate the influence of motor activity below such a threshold of MEP amplitude. We retrospectively analysed TMS‐EMG data collected during resting MT (RMT) measurement in 45 healthy control subjects (1794 data points) and 35 people with amyotrophic lateral sclerosis (ALS; 1229 data points). Trials with de‐meaned root mean squared (RMS) EMG amplitude of >10 µV throughout the 200 ms prior to stimulation were rejected. Generalised linear mixed‐effects models assessed effects of muscle activity below this rejection threshold on the probability of evoking an MEP with peak‐to‐peak amplitude of ≥50 µV. Greater sub‐rejection‐threshold activity significantly increases MEP probability in control subjects and people with ALS. Models predicted a 38%–43% increase in MEP probability when baseline RMS‐EMG amplitude increased from 1 to 9 µV. Sub‐rejection‐threshold baseline activity was significantly greater in ALS than control subjects. Below a typical rejection threshold, greater baseline RMS‐EMG amplitudes markedly increase the probability of evoking MEPs with peak‐to‐peak amplitude of ≥50 µV. Effects of sub‐rejection‐threshold muscle activity should be accounted for when comparing RMT measures, particularly between cohorts where such activity differs, such as ALS and control subjects.

## Abstract (highlights)

- **What is the central question of this study?**
  Most transcranial magnetic stimulation (TMS) studies of the corticospinal tract are performed at rest to avoid changes in motor excitability caused by muscle activation. However, it remains unreported whether very small amounts of muscle activity (levels typically considered ‘resting’) can still influence TMS‐based measures of motor excitability.
- **What is the main finding and its importance?**
  We find that, even when baseline muscle activity is <10 µV root mean squared amplitude, greater muscle activity significantly increases the likelihood of evoking a muscle response (peak‐to‐peak amplitude of >50 µV). This demonstrates that minimal muscle activation can bias ‘resting’ TMS measures of motor excitability.

## 1 INTRODUCTION

Transcranial magnetic stimulation (TMS) is frequently used to study cortical and corticospinal function and infer that there are changes in cortical excitability in various neurological and psychiatric disorders (McMackin et al., 2019). In addition, repetitive TMS is often used for therapeutic purposes (Lefaucheur et al., 2020). In both research and clinical settings, the strength of the magnetic field that constitutes an adequate intensity of stimulation is typically standardised on the basis of the resting motor threshold (RMT) of each individual. Classically, this is defined as the minimum stimulation intensity required to evoke a motor evoked potential (MEP) of ≥50 µV peak‐to‐peak amplitude in a target muscle, in 50% of trials (i.e. with a probability of 0.5) (Rossini et al., 1994).

It is well established that voluntary contraction of the target muscle increases MEP amplitude (Hess et al., 1987), decreases onset latency (Rothwell et al., 1987) and reduces amplitude variability (Kiers et al., 1993). This has been demonstrated at low levels of muscle force, such as 5% of maximum voluntary contraction (Darling et al., 2006). Furthermore, muscle contraction increases the slope of stimulus–response curves (Devanne et al., 1997) and reduces the stimulation intensity at which a motor response is first evoked (Hess et al., 1987). To ensure comparability across study populations or experimental conditions, it is therefore considered essential that the RMT and other resting MEP‐based measures are obtained while the motoneurons innervating the target muscle are not in receipt of excitatory drive. To achieve this objective, protocols used to determine the RMT (and other ‘resting’ measures) typically reject MEPs recorded when a target muscle shows prestimulus EMG activity.

Unfortunately, where studies do report monitoring of muscle relaxation during TMS, many fail to either implement or report a specific metric and/or quantitative threshold above which baseline EMG activity is considered excessive (Héroux et al., 2015). Where a metric is specified, root mean squared EMG (RMS‐EMG) amplitude in the target muscle is commonly used. In such cases, the RMS‐EMG threshold above which rest is considered violated typically ranges spans 2.5–20 µV (Calvert & Carson, 2023; Calvert et al., 2020; Castiglione & Aron, 2021; McMackin et al., 2024; Vallence et al., 2023; Wendt et al., 2023) during a brief (e.g., 100–200 ms) period immediately prior to stimulation. On occasion, much higher thresholds of 50μV (Duque & Ivry, 2009) or 100μV (Duque et al., 2010, 2012) RMS‐EMG have been used. To date, however, it has not been established whether variation in background RMS‐EMG below cut‐off thresholds typically used (e.g., 10μV) influences the probability that an MEP will be evoked. Any such influence would significantly impact the inferences that can be drawn when comparing RMTs and related ‘resting’ MEP measures between groups or conditions that differ with respect to background EMG. To exemplify this point, we consider studies that engage individuals living with amyotrophic lateral sclerosis (ALS).

ALS is a neurodegenerative disorder involving the degeneration of upper and lower motoneurons. Given that it has a wide range of presentations and is prone to misdiagnosis (Galvin et al., 2017), reliable biomarkers for early detection are urgently required. It has been proposed that TMS‐based measures have the potential to meet this need (Vucic et al., 2018). Differences between people with ALS and control subjects with respect to both single‐pulse (e.g., RMT) and paired‐pulse TMS measures [e.g., short intracortical inhibition (SICI)] suggest that those with ALS exhibit cortical hyperexcitability (McMackin et al., 2024; Tankisi et al., 2021; Zanette et al., 2002). Abnormalities in SICI appear to be present even in individuals with few clinical upper motor neuron signs (Tankisi et al., 2021). As such, it is now recommended that SICI be used as a supportive diagnostic biomarker of ALS (Turner & Group, 2022). It is, however, widely appreciated that individuals with ALS show elevated resting EMG activity (relative to control subjects) and additional distinguishing features, such as fasciculations (Liu et al., 2021). Estimates of SICI are based on either differences in MEP amplitude (conditioned vs. unconditioned, for fixed intensity protocols) or the probability of evoking MEPs of specific amplitude (for threshold tracking protocols). Given that the stimulation intensities used in both protocols are defined relative to RMT, factors that exert a systematic influence on estimation of the RMT (such as the elevated resting EMG activity characteristic of ALS), have the potential to affect measures such as SICI.

In the present study, we aimed to test the hypothesis that variation in background EMG activity below a typical rejection threshold (RMS‐EMG <10μV) is related systematically to the probability of evoking an MEP of peak‐to‐peak amplitude of ≥50 µV. This was accomplished through a retrospective analysis of data collected during the tracking of RMT in both healthy control subjects and people with ALS.

## 2 MATERIALS AND METHODS

### 2.1 Ethical approval

Ethical approval was obtained from the ethics committee of St. James's Hospital (REC reference: 2017‐02). All participants were >18 years of age and provided written consent prior to participation. All work was performed in accordance with the *Declaration of Helsinki* (2008 version (World Medical Association, 2008), which does not require pre‐registration).

### 2.2 Participants

Control participants were recruited from the community. All participants with ALS were diagnosed with possible, probable or definite ALS according to the revised El Escorial Criteria (Brooks et al., 2000). People with ALS were recruited from the Irish National ALS Clinic, Beaumont Hospital, Dublin, Ireland. Participants were excluded if they had TMS contraindications according to the revised TMS screening questionnaire (Rossi et al., 2011) or a neuromuscular or neurodegenerative disorder other than ALS. Only right‐handed participants were included, that is, those with a laterality quotient of zero or higher on the Edinburgh handedness inventory (Oldfield, 1971). Participants with a RMT > 99% of maximum stimulator output (%MSO) were excluded.

### 2.3 Experimental protocol

#### 2.3.1 EMG

Participants were seated comfortably in a sofa‐style chair with wide armrests. Their arms were positioned at an angle of 90°−120° on the armrests or in the participant's lap to improve comfort and promote muscle relaxation. Ag–AgCl electrodes (Cleartrance 1700, Conmed, Haverhill, MA, USA) were placed on the abductor pollicis brevis, spaced ∼2 cm apart in a belly–tendon montage. A reference electrode was placed on the ulnar styloid of the right wrist. Bipolar EMG was recorded from the right hand. The signal was amplified, with gain 1000, and band‐pass filtered at 10–1000 Hz using BioPac EMG100C amplifiers (Biopac Systems UK, Pershore, UK). Ambient electrical noise was removed, using either a Humbug Noise Eliminator or, equivalently, a D400 Multichannel Noise Eliminator (Digitimer Ltd, Welwyn Garden City, UK). The signal was digitised at 10 kHz using a Cambridge Electronic Design Micro1401 digitiser (CED, Cambridge, UK) and recorded using Signal software v.7.0.1 (CED, Cambridge, UK).

#### 2.3.2 TMS

Monophasic magnetic pulses were delivered across the scalp using a DuoMag MP Dual stimulator (Deymed Diagnostics, s.r.o., Hronovo, Czech Republic) equipped with a 50 mm mid‐diameter figure‐of‐eight coil. The axis of intersection between the two loops of the coil was angled 45° relative to the sagittal plane, inducing posterolateral–anteromedial current across the primary motor cortex (M1). Hereafter, we refer to this as a ‘posteroanterior’ (PA) coil orientation, in keeping with the naming convention for this orientation typically used across TMS literature (Di Lazzaro et al., 2012; Hannah & Rothwell, 2017; Tani et al., 2021). To identify the TMS ‘hotspot’, stimulator output was initially increased gradually from ∼30%MSO in 10%MSO increments 2 cm anterior and 5 cm lateral from the vertex, until an MEP was elicited in the target muscle, or until 70%MSO was reached. Thereafter, the coil was moved in ∼1 cm increments along either the anterior‐to‐posterior or medial‐to‐lateral axis from this point and the stimulus intensity increased or reduced until the position that elicited an MEP in abductor pollicis brevis at the lowest stimulation intensity was identified. To ensure consistent coil placement, even landmarks corresponding to markers on the surface of the coil were marked on a cloth cap securely taped to the participant's head (outlined and illustrated in detail by McMackin et al., 2024).

This study included TMS‐EMG data collected as part of a prior study of differences in TMS‐based measures in people with ALS compared with control subjects. Single‐pulse TMS was delivered as part of an automated threshold‐tracking protocol using maximum likelihood parameter estimation by sequential testing (PEST). The protocol, developed by Professor Friedemann Awiszus (Awiszus, 2003), uses a sigmoid‐shaped logistic function to determine the %MSO at which there is a 50% probability of eliciting an MEP with a peak‐to‐peak amplitude above a specific target. In the case of the data retrospectively included in this study, this target amplitude was either 50μV (i.e., while recording RMT) or 200μV (i.e., while recording the ‘threshold hunting target’), recorded between 15 and 50 ms after pulse delivery.

**FIGURE 1:**
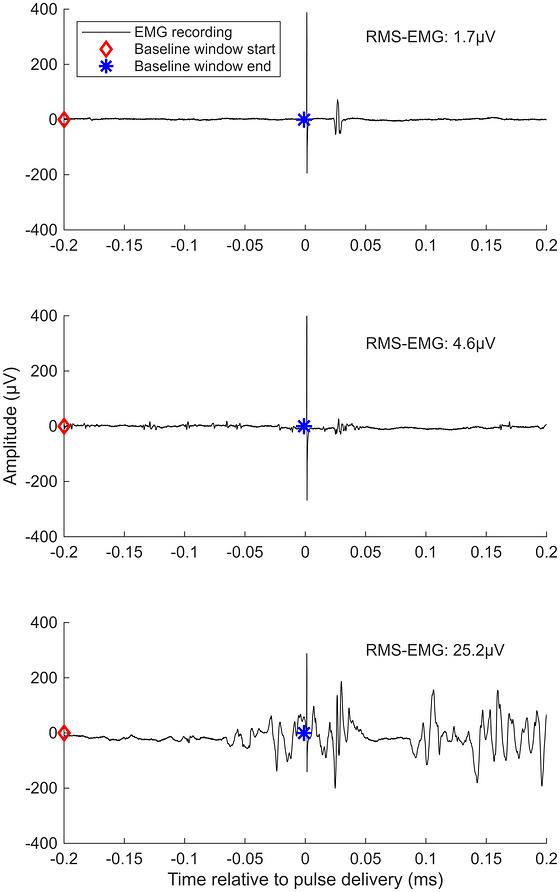
EMG recordings illustrating a range of background root mean squared EMG (RMS‐EMG) amplitudes. An artefact corresponding to the delivery of the transcranial magnetic stimulation pulse can be seen in each trace at time = 0 ms. Baseline RMS‐EMG was calculated in the window starting 200 ms prior to pulse delivery (marked by a red open diamond) ending 0.1 ms prior (marked by a blue asterisk) to pulse delivery. Data with RMS‐EMG values of >10 µV were excluded from this analysis.

A fully automatic implementation of the PEST algorithm, in MATLAB R2016a (MathWorks Inc., Natick, MA, USA) and Signal Software (CED Ltd, Cambridge, UK), was used to reduce the likelihood of human error and enable online rejection of trials with excessive baseline EMG activity, as described in detail by McMackin et al. (2024). In all cases, where RMS‐EMG in the 200 ms immediately prior to stimulation exceeded 10μV (as illustrated in Figure 1), data were not passed to the PEST algorithm, and a replacement trial was performed.

### 2.4 Data analysis

#### 2.4.1 Models

Each EMG epoch (spanning from 500 ms before to 500 ms after pulse delivery) was de‐meaned by subtracting the mean signal amplitude across the 500 ms prior to pulse delivery. Subsequently, RMS‐EMG was calculated as the RMS amplitude of EMG recorded from abductor pollicis brevis in the 200 ms immediately prior to stimulation, to align with the time window screened during online data‐quality monitoring. Our selection of a 200 ms window was based on our observations in pilot data recording that a 50 ms window was occasionally insufficient to capture evidence of muscle activity during TMS delivery. In particular, when recording data in people with ALS, such a small window can fall between ongoing fasciculations associated with lower motor neuron degeneration (Bashford et al., 2020). A longer time window was not selected because our EMG data were recorded from 500 ms prior to tracked pulse delivery, and in some sessions we were also recording long‐interval intracortical inhibition (as reported by McMackin et al., 2026). For these measures, the tracked pulse was preceded by a conditioning pulse by ≤200 ms (i.e. 300 ms after the onset of EMG capture). As such, a 200 ms window was selected for consistency across measures.

The fixed effects of baseline EMG activity (*z*‐transformed RMS‐EMG), group (person with ALS or control subject), sex and age (*z*‐transformed age in years), and their interactions, on MEP probability were assessed using binomial generalised linear mixed‐effects modelling (GLMM). Random effects of *z*‐transformed baseline EMG activity and *z*‐transformed stimulus intensity as slopes by subject identity were also examined. The goodness of fit was assessed via the Akaike information criterion and the Bayesian information criterion, and the likelihood‐ratio test (LRT) was used to assess the significance of the fixed effects and their interactions. All statistical analyses were performed in R (R Core Team, 2024), and GLMMs were implemented using the lme4 package (Bates et al., 2015).

We used a categorical response variable for the binomial GLMM, with levels of MEP/no MEP, which was equal to one if an MEP with a peak‐to‐peak amplitude ≥50 µV was evoked, or zero otherwise. Given that the data were collected as part of a prior threshold‐tracking study, stimulus intensity was not fixed within or across individuals. Correspondingly, the normalised stimulation intensity (%MSO as a percentage of RMT) applied in each trial was accounted for as part of the modelling process.

## 3 RESULTS

### 3.1 Included cohort

Data from 45 control subjects (1794 TMS‐EMG trials in total) and 35 people with ALS (1229 TMS‐EMG trials in total) were included in this study. Details of these cohorts are summarised in Table 1. Thirty participants with ALS in this study were concurrently taking riluzole, which is found to have no significant effect on RMT (Sommer et al., 1999; Stefan et al., 2001). Eight people with ALS and no control subjects were excluded from the study owing to having RMT values of >99%MSO. One control subject and two people with ALS were excluded owing to left handedness.

**TABLE 1:** Summary of statistics for amyotrophic lateral sclerosis and control cohorts.

Features	Control subjects (*n* = 45)	Subjects with ALS (*n* = 35)
Sex, female/male	17/28	11/24
Age, months, median [range]	59 [34–76]	64 [41–78]
Number of trials per participant, median [range]	39 [18–40]	40 [36–40]
**ALS‐specific features**		
Time since first symptom onset, months, median [range]	N/A	21 [7–96]
Time since diagnosis, months, median [range]	N/A	8 [1–80]
ALSFRS‐R total score^a^, median [range]	N/A	41 [17–47]
Region of onset, spinal/bulbar/respiratory	N/A	29/5/1
Site/side of first symptom onset (spinal only)^b^	N/A	3/5/4/5/8/3

Abbreviations: ALSFRS‐R, ALS functional rating scale–revised; ALS, amyotrophic lateral sclerosis; N/A, not applicable; *n*, number of participants.

^a^ ALSFRS‐R score available for ALS participants.

^b^ Spinal onset sites coded as: UL/UR/UB/LL/LR/LB (upper left/upper right/upper bilateral/lower left/lower right/lower bilateral).

### 3.2 Model selection and comparison

We found that the triple interaction among group (control subjects and people with ALS), sex and *z*‐transformed age was not significant (d.f. = 1, LRT = 2.03, *P* = 0.155). Also, there was no interaction between group and sex (d.f. = 1, LRT = 1.25, *P* = 0.263), group and *z*‐transformed age (d.f. = 1, LRT = 0.69, *P* = 0.406), and sex and *z*‐transformed age (d.f. = 1, LRT = 0.81, *P* = 0.367). Finally, there was no effect of sex (d.f. = 1, LRT = 0.06, *P* = 0.810) and *z*‐transformed age (d.f. = 1, LRT = 0.78, *P* = 0.387). As such, these effects were removed from our model. The effect of *z*‐transformed baseline EMG activity was significant (d.f. = 1, LRT = 28.80, *P* = 8.018 × 10^8^), as was the effect of group (d.f. = 1, LRT = 9.53, *P* = 0.00202) and *z*‐transformed stimulus intensity (d.f. = 1, LRT = 493.77, *P* = 2.2 × 10^16^).

The model selection based on the Akaike information criterion and the Bayesian information criterion suggested using the presented GLMM without a random intercept. This is equivalent to assuming that all participants had a similar probability of evoking an MEP with a peak‐to‐peak amplitude of ≥50 µV when all predictors are set to zero (i.e., their mean value, because all predictors were *z*‐transformed during model fitting). The low variance of the intercept is likely to be caused by the normalisation of stimulus intensity to the RMT for each participant. By definition, RMT is the stimulation intensity at which there is a 50% probability of evoking an MEP with peak‐to‐peak amplitude of ≥50 µV. Therefore, at a fixed value of stimulation intensity, MEP probability is likely to be similar across participants. Overall, each binary response $y_{ij}$ for an observation i from a subject j follows a Binomial distribution:

$$y_{ij}∼\mathrm{Binomial}\left(1,\pi_{ij}\right)$$

where $\pi_{ij}=P\left(\;\mathrm{MepOccurre}d_{ij}=1\right)$ is the probability of an MEP occurring for observation i belonging to subject j. The following logit link function was used:

$$\mathrm{logit}\;\left(\pi_{ij}\right)=\log\left(\frac{\pi_{ij}}{1-\pi_{ij}}\right)={\hat{\eta }}_{ij}$$

where:

$${\hat{\eta }}_{ij}=\underset{\mathrm{Fixed}\;\mathrm{effects}}{\underset{︸}{{-16.6+0.42\times \mathrm{RM}S_{ij}-0.36\times {\left[\mathrm{ALS}\right]}_{j}+16.9\times \mathrm{Intensity}}_{ij}}}+\underset{\mathrm{Random}\;\mathrm{effects}}{\underset{︸}{{u_{1j}\times \mathrm{RM}S_{ij}+u_{2j}\times \mathrm{Intensity}}_{ij}}}$$

where:

- 95% confidence interval for the intercept of model's is [–18.36, –14.92];
- $\mathrm{RM}S_{ij}$ = *z*‐transformed RMS baseline EMG activity (95% confidence interval: [0.27, 0.59]);
- ${\left[\mathrm{ALS}\right]}_{j}$ = indicator variable equal to one if subject j has a diagnosis of ALS, zero if a control subject (95% confidence interval: [–0.59, –0.13]); and
- $\mathrm{Intensit}y_{ij}$ = *z*‐transformed stimulus intensity (95% confidence interval: [15.26, 18.65]).

**FIGURE 2:**
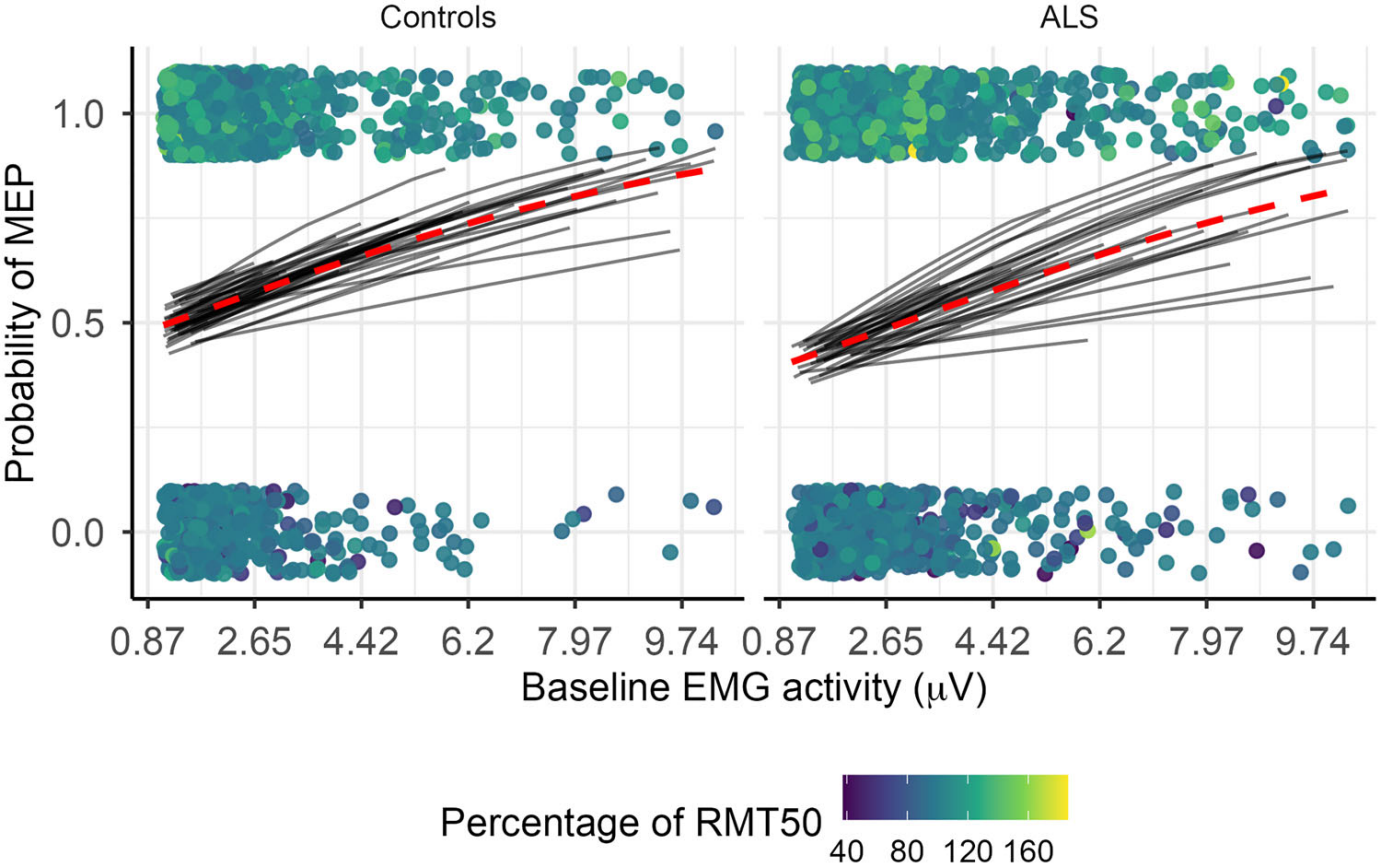
Predicted values for MEP probability, when stimulation intensity is 100% of resting motor threshold (RMT50), across baseline amplitude values. The red dashed line represents the average model‐predicted probability of evoking an MEP with a peak‐to‐peak amplitude of ≥50 µV. Individual black lines represent the model‐predicted probability by subject. Baseline EMG activity is the root mean squared EMG amplitude recorded in the 200 ms immediately prior to pulse delivery. The *x*‐axis tick labels are rounded to two decimal places following inverse transformation of modelled *z*‐transformed baseline EMG activity values. Coloured points represent individual transcranial magnetic stimulation trials, in which an MEP either occurred (top) or did not occur (bottom) for 45 controls (*n* = 1794) and 35 people with ALS (*n* = 1229). Data points are vertically jittered to limit overlap, with colour indicating stimulus intensity as a percentage of RMT50 for each individual. Abbreviations: ALS, amyotrophic lateral sclerosis; MEP, motor evoked potential; RMT, resting motor threshold.

**FIGURE 3:**
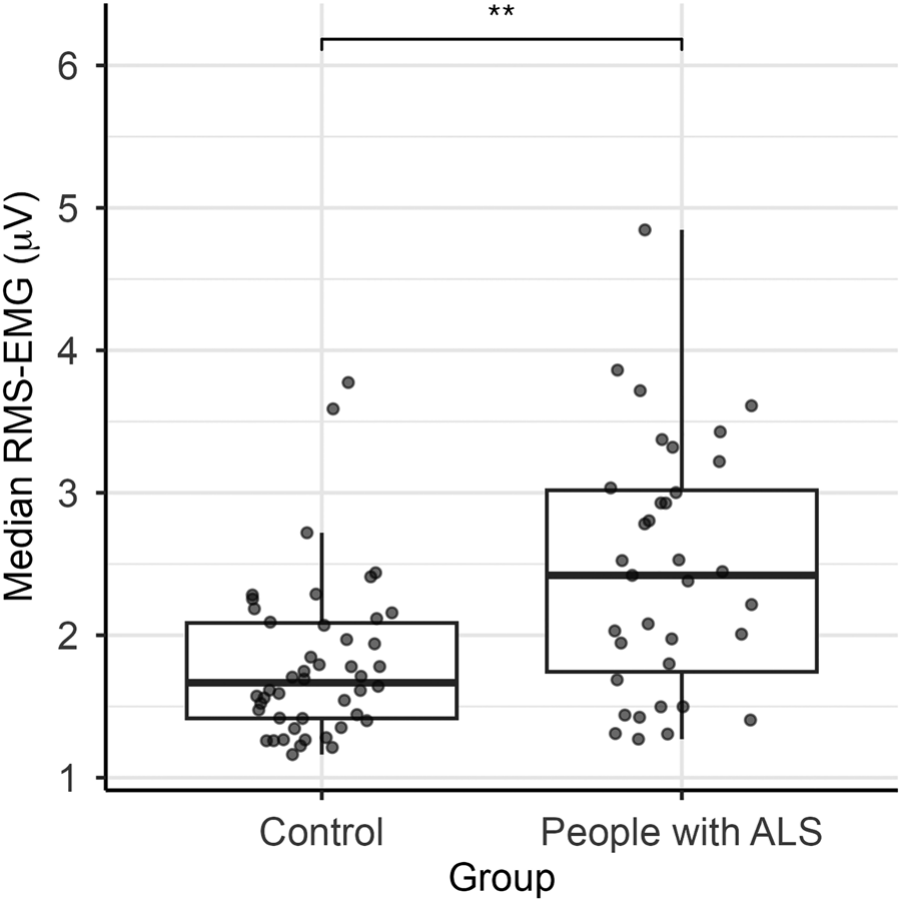
Comparison of median RMS‐EMG amplitudes in control subjects and people with ALS across all trials. Boxplots display the mean and interquartile range. Whiskers extend to the largest value no greater than 1.5 times the interquartile range. **P = 0.00202 for likelihood ratio test of modelled group effect. Abbreviations: ALS, amyotrophic lateral sclerosis; RMS, root mean square.

To illustrate our findings, Table 2 presents the predicted probability of evoking an MEP of peak‐to‐peak amplitude ≥50 µV for selected baseline RMS‐EMG values, where stimulus intensity is 100% of RMT. Predicted MEP probabilities across the modelled range of baseline RMS‐EMG values for each individual, in addition to the average prediction for each group, are also illustrated in Figure 2. Finally, the distribution of median RMS‐EMG values for each participant is illustrated in Figure 3, to illustrate the significant increase in baseline activity observed in people with ALS compared with control subjects (d.f. = 1, LRT = 9.53, *P* = 0.00202).

**TABLE 2:** Model predicted values and 95% confidence interval (in parentheses) for motor evoked potential probability when stimulus intensity is 100% RMT, across three selected integer values of RMS‐EMG within the modelled range.

RMS‐EMG (µV)	Control subjects	Subjects with ALS
1	0.49 (0.44, 0.53)	0.40 (0.34, 0.44)
5	0.72 (0.66, 0.77)	0.64 (0.57, 0.70)
9	0.87 (0.79, 0.92)	0.83 (0.73, 0.89)

Abbreviations: ALS, amyotrophic lateral sclerosis; RMS, root mean square; RMT, resting motor threshold.

## 4 DISCUSSION

In this study, we have demonstrated that, within the range of 0–10 µV, variations in background RMS‐EMG amplitude systematically influence the probability of evoking an MEP of ≥50 µV. Moreover, the size of this effect is non‐negligible; in healthy control subjects, an increase in RMS‐EMG activity from 1 to 9μV results in an increase of > 27% in the probability of evoking an MEP that meets the standard 50 µV amplitude criterion. An increase from 1 to 5μV corresponded to an increase in probability of 23%. We found that this effect could be replicated in data from a cohort of people with ALS, with increase in RMS‐EMG activity from 1 to 9μV resulting in a 43% increase in MEP probability and an increase from 1 to 5μV corresponded to a 24% increase in MEP probability. Thus, our findings indicate that estimated RMT values are likely to vary inversely with background muscle activity, even when trials with RMS‐EMG of ≥50 µV are excluded.

To our knowledge, no other study has explicitly modelled the effects of variations in RMS‐EMG below 10μV. The findings have important implications for research that uses TMS to study motor networks in health and disease. Currently, there is no standard procedure to control for the presence of background muscle activity. Baseline EMG amplitude rejection thresholds vary significantly from study to study and can range from 2.5 (Calvert & Carson, 2023) to 100μV (Duque et al., 2010, 2012). In addition, many studies do not report a rejection threshold or the time window within which background EMG is monitored (Héroux et al., 2015). Several investigators seek to verify relaxation simply by online visual or auditory monitoring of the EMG.

These findings have important implications for the definition and recording of RMT. RMT is defined as the TMS stimulus intensity for which there is a 50% probability of evoking an MEP at rest. However, an important assumption of threshold estimation, including via the PEST‐based approach used to estimate RMT here, is that MEP probability is constant for a given stimulus strength. Our modelling shows that even for the range of RMS‐EMG values within which a muscle is defined as ‘resting’, there is significant variation in MEP probability. This is supported by previous findings of state‐dependent changes in RMT, for example, during motor imagery (Karabanov et al., 2015). Therefore, the RMT measures we have used to normalise stimulation intensity across individuals in our model might not reflect motor threshold at complete rest.

In most threshold tracking algorithms, including the PEST protocol used here, threshold estimates are continuously refined over successive trials. As such, random fluctuations in very low‐amplitude baseline EMG across trials are unlikely to have a profound effect on tracked TMS measures within an individual. However, the identified influence of such low‐level activity could act as an important variable where average resting amplitudes vary systematically between compared populations or repeated measurements. As such, it is particularly important to consider the potential effects of EMG activity that is below the nominal rejection threshold when a study involves people with neurological disorders, such as ALS, that affect motoneuron excitability. Such systematic deviations in ‘resting’ baseline activity are likely to exist when comparing people with and without ALS or when comparing people with ALS at different stages of functional decline. We have demonstrated that in a sample of people with ALS, background EMG in trials that would typically be deemed acceptable (i.e. background RMS‐EMG ≤10 µV) is significantly greater than in control subjects. Consequently, investigators should assess carefully how the effects of such differences in background muscle activity can be mitigated when using outcome measures based on MEP amplitudes.

Notably, the model reported here is specific to the hardware used to collect these data. For example, our data collection includes the removal of electrical noise through the use of either a Humbug Noise Eliminator or equivalently, a D400 Multichannel Noise Eliminator (Digitimer Ltd, Welwyn Garden City, UK). In laboratories where these or other methods are not used to remove such noise, higher baseline EMG amplitudes might reflect environmental factors that do not affect MEP amplitude. As such, reports of higher baseline EMG amplitudes in prior literature might not necessarily represent target muscle activation. In future, when such environmental noise cannot be eliminated, additional steps must be taken to report and account for background muscle activity in the presence of such noise (such as through application of appropriate notch filters prior to further data screening and analysis).

Our modelling (see Figure 2) suggests that the effect of variations in background muscle activity on MEP amplitudes is likely to be small for levels of RMS‐EMG of <2 μV. In practice, however, the adoption of this stringent criterion (particularly for people with neurological disorders) will probably lead to extensive rejection and repetition of trials, thus prolonging the required duration of recording sessions. This can often be a disincentive to participation and can induce fatigue or further muscle tension during recording. An alternative would be to include RMS‐EMG as a covariate in statistical models, with the aim of accounting for the influence of its variation on outcome measures. Alternatively, studies using protocols dependent upon RMT could continuously monitor RMT and suitably account for fluctuations in the degree of ‘rest’ during recording. Such an approach has been adopted in some threshold tracking TMS studies of ALS (Jacobsen et al., 2024; Tankisi et al., 2021) and in a recent fixed‐intensity TMS study in control subjects (Fanella et al., 2025). In these studies, the estimated RMT, and conditioning stimulus intensities dependent thereon, are adjusted continuously to account for fluctuations in cortical excitability (Tankisi et al., 2023). At a minimum, in all TMS‐EMG‐based studies, investigators should clearly state: (1) the time window in which prestimulation muscle activity is registered; (2) the specific threshold of baseline EMG activity above which trials will be rejected (following implementation of measures to eliminate environmental noise); and (3) the presence of any differences in EMG activity between experimental conditions or cohorts. In this regard, inferential tests of equivalence should be used.

## AUTHOR CONTRIBUTIONS

Roisin McMackin: Conceptualization; Séamus O'Sullivan, Roisin McMackin, Cathal Walsh, Gabriel R. Palma: Methodology; Friedemann Awiszus, Gabriel R. Palma: Software; Séamus O'Sullivan, Roisin McMackin, Gabriel R. Palma: Formal analysis; Orla Hardiman: Resources; Roisin McMackin, Yasmine Tadjine, Narin Suleyman, Eva Woods, Antonio Fasano: Investigation; Roisin McMackin: Data curation, Supervision, Project Administration; Séamus O'Sullivan, Roisin McMackin, Gabriel R. Palma: Writing—Original Draft; Séamus O'Sullivan, Roisin McMackin, Yasmine Tadjine, Narin Suleyman, Eva Woods, Antonio Fasano, Cathal Walsh, Friedemann Awiszus, Orla Hardiman, Richard G. Carson, Gabriel R. Palma: Writing—Review & Editing; Séamus O'Sullivan, Gabriel R. Palma: Visualisation; Roisin McMackin, Richard G. Carson, Orla Hardiman: Funding Acquisition. All authors approved the final version of the manuscript and agree to be accountable for all aspects of the work in ensuring that questions related to the accuracy or integrity of any part of the work are appropriately investigated and resolved. All persons designated as authors qualify for authorship, and all those who qualify for authorship are listed.

## CONFLICT OF INTEREST

None declared.

## GENERATIVE AI STATEMENT

No generative AI tools were used in the preparation of this manuscript.

## Data Availability

Individual datasets are not at present openly accessible for compliance with General Data Protection Regulation, as data remain pseudonymised. Data may be shared upon reasonable request and completion of a suitable data sharing agreement. Summary statistics used to produce Figure 2 and 3 are available at https://doi.org/10.5281/zenodo.21037905.
